# Regulatory T Cells in Autoimmune Vasculitis

**DOI:** 10.3389/fimmu.2022.844300

**Published:** 2022-02-28

**Authors:** Ke Jin, Simon Parreau, Kenneth J. Warrington, Matthew J. Koster, Gerald J. Berry, Jörg J. Goronzy, Cornelia M. Weyand

**Affiliations:** ^1^ Department of Medicine, Mayo College of Medicine and Science, Rochester, MN, United States; ^2^ Department of Pathology, Stanford University School of Medicine, Stanford, CA, United States; ^3^ Department of Medicine, Stanford University School of Medicine, Stanford, CA, United States

**Keywords:** T cells, vasculitis, giant cell arteritis, Treg cell, exosomes, intracellular vesicles, NOTCH, autoimmune disease

## Abstract

Blood vessels are indispensable for host survival and are protected from inappropriate inflammation by immune privilege. This protection is lost in patients with autoimmune vasculitides, a heterogeneous group of diseases causing damage to arteries, arterioles, and capillaries. Vasculitis leads to vascular wall destruction and/or luminal occlusion, resulting in hemorrhage and tissue ischemia. Failure in the quantity and quality of immunosuppressive regulatory T cells (Treg) has been implicated in the breakdown of the vascular immune privilege. Emerging data suggest that Treg deficiencies are disease-specific, affecting distinct pathways in distinct vasculitides. Mechanistic studies have identified faulty CD8^+^ Tregs in Giant Cell Arteritis (GCA), a vasculitis of the aorta and the large aortic branch vessels. Specifically, aberrant signaling through the NOTCH4 receptor expressed on CD8^+^ Treg cells leads to rerouting of intracellular vesicle trafficking and failure in the release of immunosuppressive exosomes, ultimately boosting inflammatory attack to medium and large arteries. In Kawasaki’s disease, a medium vessel vasculitis targeting the coronary arteries, aberrant expression of miR-155 and dysregulated STAT5 signaling have been implicated in undermining CD4^+^ Treg function. Explorations of mechanisms leading to insufficient immunosuppression and uncontrolled vascular inflammation hold the promise to discover novel therapeutic interventions that could potentially restore the immune privilege of blood vessels and pave the way for urgently needed innovations in vasculitis management.

## Introduction

Vasculitides are autoimmune diseases defined by tissue-destructive inflammation in the vessel wall, resulting in wall destruction or wall remodeling leading to alterations in the vascular lumen. The pathogenic remodeling of blood vessels restricts the supply of nutrients and oxygen to peripheral tissues, causing organ damage and death (1). Vasculitides can be classified into diverse types according to the size of the affected vessels (2) and share some common phenotypes, such as wall infiltration of inflammatory cells, aneurysm formation, and luminal compromise (3). Decades of studies have yielded insights into the various mechanisms underlying the autoimmune inflammation of arteries. Arteries are also the target of uncontrolled inflammation in auto-inflammatory syndromes, emphasizing the delicate relationship between immune cells and the conduits that carry them to the peripheral tissues (4).

A more detailed understanding of the immunopathogenesis of vasculitis is available for Giant Cell Arteritis (GCA), an inflammatory vasculopathy of medium and large arteries. In GCA, aberrant NOTCH signaling appears to have high pathogenic relevance and contributes to the breakdown of the immune privilege (5, 6). Inappropriate production of MMP9 by constituent myeloid cells adds another element to the loss of the tissue barrier (7). Additional pathways of pathogenic relevance include a state of hypermetabolism imposed by excessive CD28 signaling (8), the loss-of-function of the immunosuppressive PD1/PDL1 checkpoint (9), and the longevity of tissue-resident memory T cells that sustain chronic inflammation (10). This panel of malfunctioning pro-inflammatory pathways is complemented by the failure of anti-inflammatory mechanisms. Specifically, CD8^+^ Treg cells fail to provide proper inhibitory function in GCA patients (11–13). CD8^+^ Treg cells exert their immunosuppressive role by packaging NADPH2 oxidase 2 (NOX2) into exosomes and releasing these exosomes to control the function of neighboring T cells. In GCA patients, exosomal NOX2 is low, due to a defect of directing intracellular vesicles. The rerouting of vesicles is a consequence of inappropriate signaling through the NOTCH4 receptor (12, 13).

## Regulatory T (Treg) Cells

Regulatory T (Treg) cells are a subset of T lymphocytes, occupying about 5% to 10% of the circulating T cell pool (14). In contrast to effector T cells, Treg cells mediate immunosuppression to ensure that the immune defenses against exogenous and endogenous antigens are accurately controlled in time and space. Treg cells accomplish their suppressive function through numerous mechanisms, such as secretion of inhibitory cytokines, induction of apoptotic cell death, direct transfer of inhibitory signals, and the delivery of extracellular vesicles (15–17).

A shift in either quantity or quality of Treg cells will lead to a disbalance in immune homeostasis, resulting in a variety of disease states. In tumor-bearing hosts, Treg cells are enriched at the tumor site, suppress anticancer immunity, protect the tumor from immunosurveillance and promote tumor development (18, 19). In contrast, patients with autoimmune disease, including vasculitis, suffer from defective Treg cell protection, promoting a breakdown of tissue tolerance and a lack of timely downregulation in ongoing immune responses (20, 21). Accordingly, massive efforts have been undertaken to turn Treg cells into therapeutic agents or enhance Treg function in patients. These investments have resulted in the development of novel Treg-based therapeutic strategies, which will eventually provide novel alternatives for immune-modulatory interventions (22, 23). In this review, we will summarize current knowledge of the phenotypes of Treg cells, their protective roles in vasculitis, and potential strategies for harnessing Treg cell function.

### Treg Phenotypes

Here, we will provide an overview of what is known about shared phenotypes found on most Treg cells, including the Treg cell marker (FOXP3), specific subtypes of Treg cells, e.g. CD8^+^ Treg cells, and the molecular mechanism behind Treg-induced inhibition. There is agreement in the field that Treg cell populations are heterogenous when it comes to phenotype and function (24). In addition, there is recognition that Treg cells have considerable plasticity, which may be particularly important when they are exposed to inflammatory signals (25–27). Multiple factors, including the tissue environment in which the Treg cell lives and functions, the extent of antigen-induced T cell receptor signalling, the input of co-stimulatory and co-inhibitory signalling may all contribute to the stability and the plasticity of Treg cells.

#### FOXP3

The transcription factor forkhead box P3 (FOXP3) is the best known Treg biomarker and is recognized as the master regulator of Treg cell generation and function. As a transcription factor, FOXP3 induces the expression of Treg-associated genes, including IL-2, CD25, CTLA-4, and miR-155 (28, 29). More than a recognitional marker, FOXP3 possesses the ability to control the switch between Treg cells and effector T cells. Ectopic FOXP3 expression transforms T cells into suppressor cells, while the failure of constant FOXP3 expression impairs the potency to inhibit effector cells (30–32). Based on FOXP3 expression, Treg cells can be dissected into three subpopulations: CD45RA(+)FOXP3(lo) resting Treg cells (rTreg cells), CD45RA(-)FOXP3(hi) activated Treg cells (aTreg cells) and cytokine-secreting CD45RA(-)FOXP3(lo) nonsuppressive T cells. Both rTreg cells and aTreg cells are effective suppressor cells *in vitro*. aTreg cells die rapidly but rTreg cells proliferate and convert into aTreg cells (24).

Due to FOXP3’s indispensable role in Treg biology, the regulation of FOXP3 expression has drawn considerable attention. Several transcription factors have been reported to induce FOXP3 transcription, including Forkhead transcription factor of the O class (FOXO)1, FOXO3, c-Rel, Smad2, and Smad3 (33–39). Interestingly, the glycolytic enzyme enolase (ENO)-1 inhibits FOXP3 transcription through binding to the promoter region (40), suggesting a major role in the direct metabolic control of Treg cell function. At the post-transcriptional stage, several microRNAs are predicted to directly bind to the FOXP3 3’-UTR. Specifically, changes in the abundance of miR-31 and miR-15a/16 have been associated with significant modulation of FOXP3 expression (41–44). FOXP3 can also be regulated through post-translational modifications. High expression of the deubiquitinase (DUB) ubiquitin specific-processing protease (USP)7 in Treg cells is required for sustained FOXP3 expression (45). Vice versa, the E3 ubiquitin ligase Stub1 leads to FOXP3 ubiquitination and degradation (46, 47). Uncontrolled FOXP3 ubiquitination has been proposed as a relevant mechanism in autoimmune diseases. In patients with psoriasis, the (C-C motif) ligand (CCL)3 and the protein kinase B (PKB)a/Akt1 pathway induce polyubiquitination of FOXP3, which is highly associated with defective Treg function (48). Besides ubiquitination, FOXP3 is also regulated by other modifications, including acetylation (by SIRT1 and TIP60) and phosphorylation (by CDK2 and NLK) (49–54). In the autoimmune disease rheumatoid arthritis (RA), instability of FOXP3 expression and the subsequent failure of Treg-dependent immunosuppression are due to the insufficient expression of the histone acetyltransferase TIP60 (55).

#### CD8^+^ Treg Cells

Although CD25^+^FOXP3^+^ T cells amongst CD4^+^ T cells are often considered to be classical Treg cells, the compartment of CD8^+^ T cells also contains a regulatory subset. Like CD4^+^ Treg cells, CD8^+^ Treg cells express FOXP3, but at a lower level (56, 57). Recent studies have emphasized that it is not accurate to identify CD8^+^ Treg cells based only on CD25 expression (58, 59). Instead, additional cell surface markers, such as CD39^+^ and CD26^-^ are now considered useful markers of CD8^+^ Treg cells (60, 61). The subpopulation of CD8^+^CD39^+^CD26^-^ T cells represents a highly purified Treg cell subset, which possesses strong inhibitory effects in T cell activation assays (12).

Similar to CD4^+^ Treg cells, CD8^+^ Treg cells may be reduced in number or quality in patients with autoimmune disease. Specifically, lowered numbers of CD8^+^ Treg cells have been reported in patients with systemic lupus erythematosus (SLE) and recovery of CD8^+^FOXP3^+^ Treg cells after transplantation of autologous hematopoietic progenitor cells has been associated with good control of disease activity (62). In patients with giant cell arteritis (GCA), frequencies of circulating CD8^+^ Treg cells are largely maintained, but an altered gene expression program results in impaired suppressive capacity and unopposed inflammatory activity of pathogenic CD4^+^ T cells. Experiments designed to repair the expression of relevant gene products in patient-derived CD8^+^ Tregs have been sufficient to restore fully functional Tregs *in vitro* and *in vivo*, which prevented the invasion of the vessel wall by inflammatory cells (13).

#### Mechanisms of Treg Cell Function

**Figure 1:**
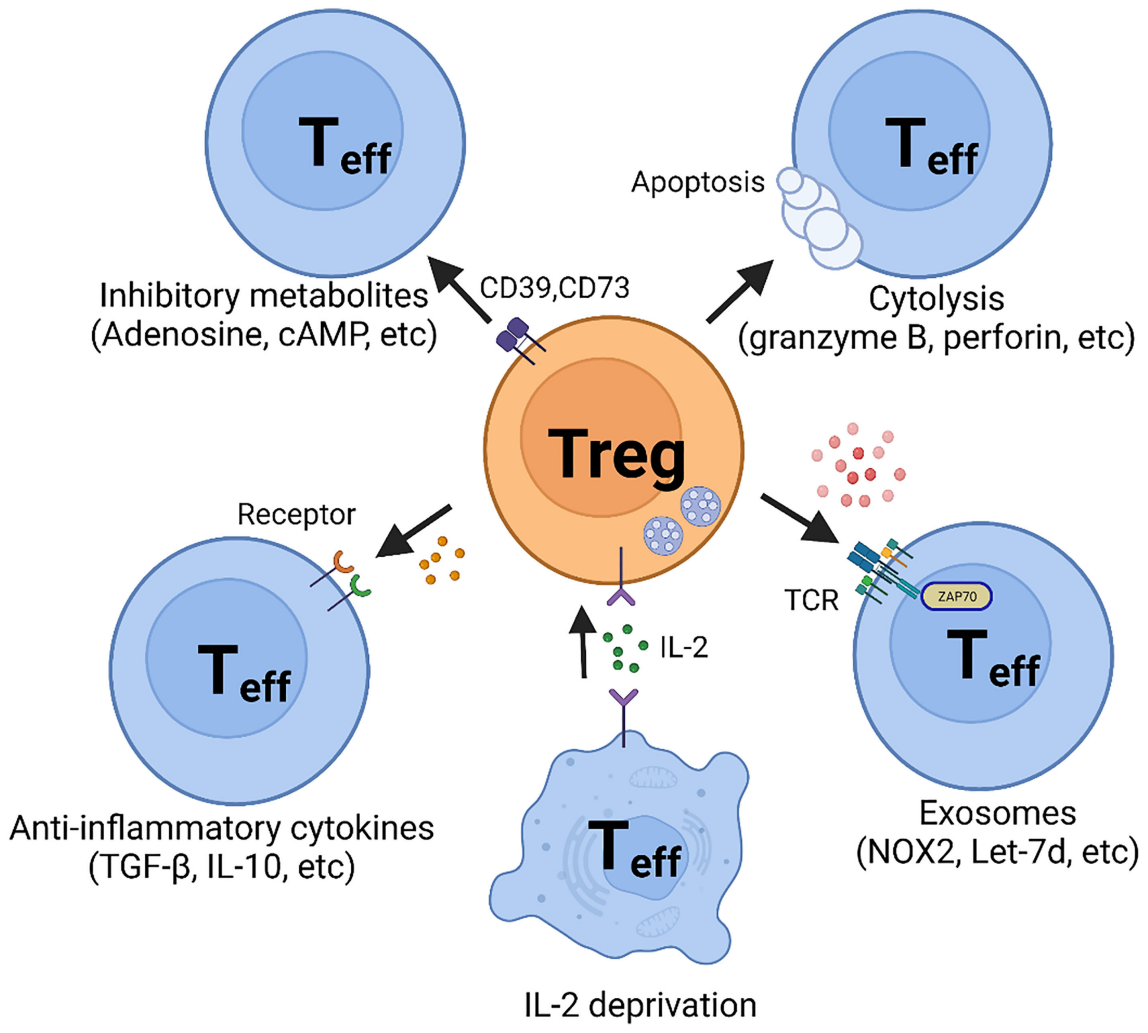
Suppressive Mechanisms of regulatory T (Treg) cells. Regulatory T cells perform suppressive function mainly through five basic mechanisms: 1. Inhibitory cytokines, such as TGF-β and IL-10; 2. Metabolic disruption, including CD39- and CD73-generated adenosine and gap junction-mediated cAMP delivery; 3. Cytolysis, utilizing granzyme B- and perforin- dependent mechanisms; 4. Exosomal delivery of immunosuppressive biomolecules, such as NOX2 and Let-7; 5. Competition for IL-2.

Determination of the mechanisms of how Treg cells function is critical to understanding the role of these specialized T cells in protective and pathogenic immunity. Ever since Treg cells were initially described, attention has been directed at uncovering, both on the cellular and molecular level, how these cells can inhibit signaling to affect the survival of their target cells. Here, we are going to summarize the spectrum of mechanisms used by Treg cells to exert their immune-regulatory role (**Figure 1**).

##### Cytokines

Inhibitory cytokines secretion may be one of the most efficient mechanisms of Treg cell-mediated suppression of immune responses, but this is complicated by nonselectivity. Although multiple inhibitory cytokines have been discovered, interleukin-10 (IL-10) and Transforming growth factor-β (TGF-β) remain on top of the list and are considered part of the basic repertoire in Treg cell biology (**Figure 1**).

The IL-10 family has nine members, including IL-10, IL-19, IL-20, IL-22, IL-24, IL-26, IL-28A, IL-28B, and IL-29 (63) and is now recognized as a critical element in protecting tissues from excessive inflammatory responses (64). Upon binding to its receptors IL-10R1 and IL-10R2, IL-10 regulates downstream pathways through cascade phosphorylation, utilizing the JAK-STAT signaling pathway (65, 66). IL-10-dependent activation of the JAK-STAT signaling pathway leads to nuclear translocation of STAT3, initiating downstream target gene expression (67, 68). IL-10 has also been reported to activate other signaling pathways, such as PI3K-AKT and MAPK pathways (69–72). Abnormal expression of IL-10 has been reported in some vasculitides. Tesar et al. found that IL-10 is highly expressed in patients with active ANCA-associated vasculitis (AAV), but not in patients in remission (73). The upregulation of IL-10 with disease activity might be reflective of the host’s attempt to suppress inappropriate immunity. The authors also reported that patients in remission who then relapsed produced significantly lower levels of IL-10 compared to those without relapse, indicating that IL-10 could be a useful biomarker for long-term disease prediction (73).

TGF-β mediates its anti-inflammatory function through two routes: 1. Inhibition of inflammatory cells; 2. strengthening of Treg cells (74). TGF-β has been reported to suppress T cell proliferation by blocking production of interleukin-2 (IL-2) (75) and preventing T cell differentiation to Th1 and Th17 cells (76–78). In addition, TGF-β promotes the generation and function of Treg cells by inducing FOXP3 expression in both CD4^+^ and CD8^+^ T cells (79–81). Although TGF-β has three isoforms, TGF-β1 is dominantly expressed in the immune system (82). Twelve TGF-β receptors with serine/threonine kinase subunits have been enumerated, including 5 type I and 7 type II transmembrane receptors (83). Upon TGF-β stimulation, the signaling cascade of SMAD proteins causes the transcription of target genes, such as MYC and P21 (84, 85). Other non-SMAD pathways have also been implicated in TGF-β signaling, such as the RAS-ERK pathway and the PI3K-AKT pathway (86).

An alternative mechanism through which Treg cells modulate the function and behavior of T effector cells relates to the competition for resources. Specifically, Treg cells can inhibit the proliferation and survival of effector T cells by depriving them of IL-2 (87).

##### Inhibition Through the Transfer of Metabolites

Data have accumulated supporting the concept that metabolites generated and released by Treg cells impose an inhibitory effect on targeted T cells.

Adenosine, generated by CD39 and CD73 in the extracellular space or released from the intracellular compartment, suppresses effector T cells by binding to the adenosine receptor 2A (A_2A_R) (88–90) (**Figure 1**). Evidence has been provided that adenosine promotes the generation of Treg cells by inhibiting IL-6 generation and enhancing TGF-β production. In mice, A_2A_R stimulation results in the inhibition of Th1 and Th17 cell differentiation and the enhancement of FOXP3^+^ Treg cell generation (91). No data are available on whether this mechanism has relevance in inflammatory vasculopathies. Notably, methotrexate, an immunosuppressive medication used broadly in the treatment of autoimmune diseases by amplifying adenosine production, has only limited application in patients with autoimmune vasculitis.

Alternative pathways through which Treg cells control the functionality of effector T cells involve cyclic AMP (cAMP) (**Figure 1**). Treg cells harbor a high concentration of cAMP and form a cell contact-dependent gap junction with effector T cells to deliver intracellular cAMP (92). Subsequently, cAMP inhibits T cell proliferation and IL-2 synthesis (92).

##### Cytolysis

An alternative mode through which Treg cells inflict their suppressive function relies on perforin/granzyme-dependent cytolysis (**Figure 1**). Although cytotoxicity mediated by perforin/granzyme has mostly been considered an exclusive function of natural killer (NK) cells and CD8^+^ T cells, it is now accepted that Treg cells and CD4^+^ T cells can utilize this mechanism to regulate the function of neighboring cells. Recent studies have provided convincing evidence that some human CD4^+^ T cells also exhibit cytolytic function (93, 94). Granzyme B has been detected in CD4^+^ Treg cells and has been associated with functional fitness. Granzyme B appears to be highly expressed in CD4^+^FOXP3^+^ Treg cells and deficiency for granzyme B or perforin paralyzed Treg cells in a mouse model (95).

##### Exosomal Delivery

Exosomes are extracellular vesicles secreted by cells, with sizes ranging from roughly 50 – 150 nm. Exosomes contain cell-specific proteins, lipids, metabolites, and genetic materials, and can be selectively absorbed by neighboring or distant cells based on surface protein recognition (96, 97). Exosomes play a critical role in immune regulation but seem to be of special relevance for Treg cells since Treg cells outperform other cell types in the production of exosomes. Treg-derived exosomes are indispensable for Treg cell function and disruption of exosome release through pharmaceutical or genetic mechanisms effectively blocks the suppressive function of Treg cells (16, 98, 99).

Exosomes exhibit their function through the biomaterial they contain, such as lncRNA (e.g., Let-7d) (100) or inhibitory molecules (e.g., CD73) (101). In the large vessel vasculitis, GCA, CD8^+^ Treg cells inhibit CD4^+^ T cells activation and proliferation by secreting NADPH oxidase 2 (NOX2) containing exosomes (12, 13) (**Figure 1**). Blocking exosome secretion or interfering with NOX2 function halted CD8^+^ Treg cell function. Enhancing the loading of NOX2 into the exosome restored CD8^+^ Treg cells’ function and attenuated vascular inflammation in chimeric mice engrafted with human arteries. These data support the concept that Treg-derived exosomes may provide a novel tool to reestablish tissue tolerance in vasculitis (12, 13).

### Treg Cells in Autoimmune Vasculitides

#### Giant Cell Arteritis

Giant Cell Arteritis (GCA) is the most common autoimmune vasculitis, with incidence and prevalence rates growing as the population ages. Patients with GCA suffer from aggressive wall inflammation in medium and large arteries, including the aorta, subclavian arteries, axillary arteries, extra-cranial branches of the carotid arteries, and vertebral arteries. Infrequently, GCA is diagnosed in lower extremity arteries. Clinically, the most feared disease manifestations are ischemic stroke of the optic nerve and the posterior brain. Potentially fatal consequences such as aneurysm formation, dissection and rupture are related to the destruction of the aortic wall. On a cellular level, the disease is characterized by the formation of granulomas within inflamed arteries, assembled from CD4^+^ T cells, macrophages and multinucleated giant cells (102, 103).

Numeric and qualitative deficiencies in Treg cells are well recognized in GCA. Terrier and colleagues reported decreased frequencies of CD4^+^FOXP3^+^ Treg cells in the peripheral blood of GCA patients and FOXP3 was suspiciously absent in T cells infiltrating vasculitic lesions (104).

**Figure 2:**
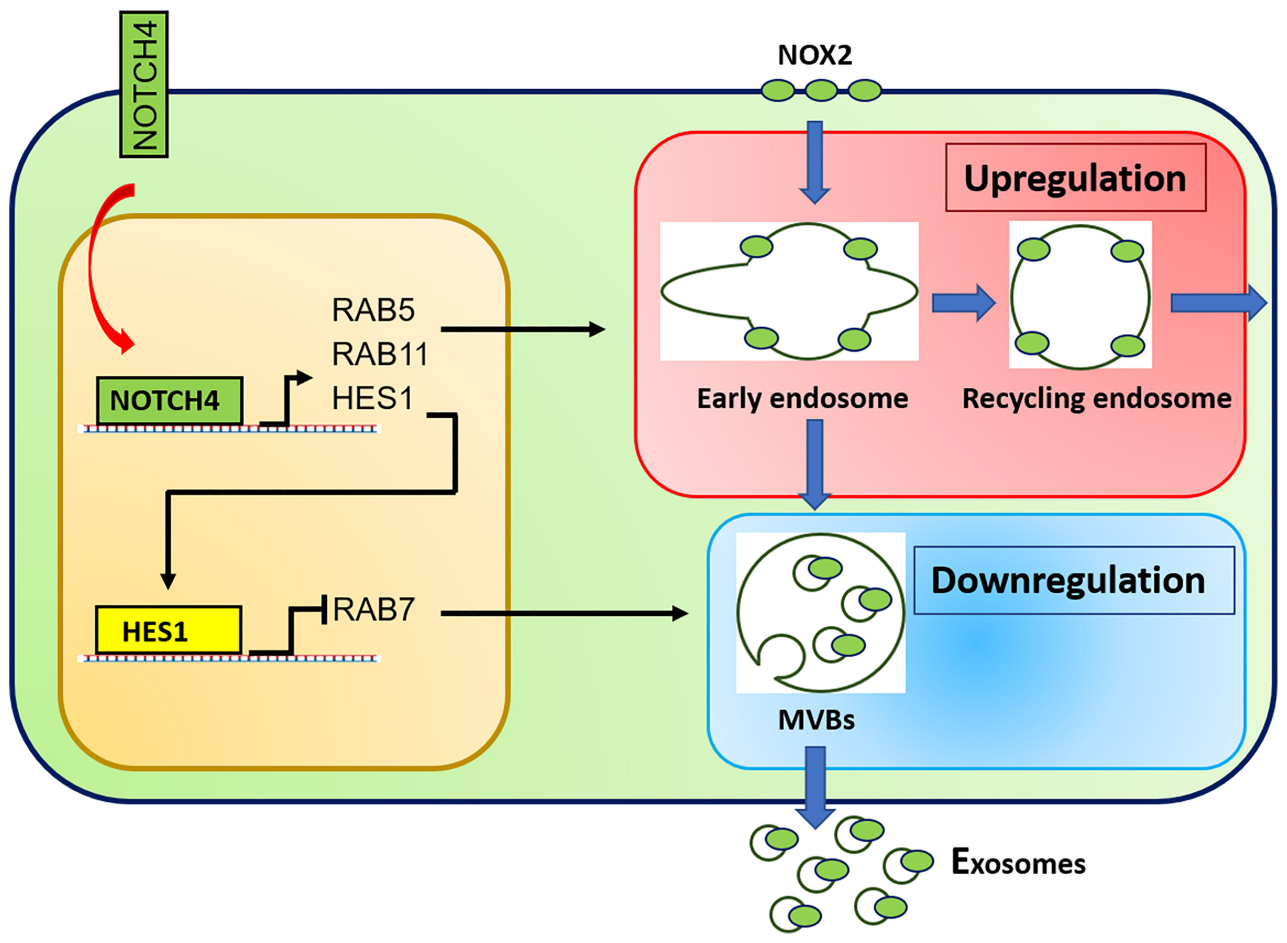
Molecular defects in CD8^+^ Treg cells from GCA patients. Aberrant NOTCH4 signaling in GCA CD8^+^ Treg cells enhances expression of *RAB5A* and *RAB11A* and suppresses expression of *RAB7A* through HES1. *RAB5A* and *RAB11A* high expression promotes formation of early and recycling endosomes, keeping NOX2 in an intracellular, non-secretory storage compartment. *RAB11* suppression results in deficient generation of specialized endosomes, the multivesicular bodies (MVB) and subsequent reduction in exosome biogenesis. Consequently, NOX2 is trapped intracellularly and no longer available to be loaded into immunosuppressive exosomes.

Recent cellular and molecular studies have shifted attention from CD4^+^ Treg cells to the CD8^+^ Treg cell subset. CD8^+^ Treg cells contain a functionally specialized subpopulation that imposes immunosuppression by secreting NOX2 containing exosomes that are absorbed by nearby CD4^+^ effector T cells to suppress their activation and proliferative expansion (12). In patients with GCA, the frequency of CD8^+^ NOX2^+^ Treg cells is diminished, and, in addition, their function is essentially paralyzed. Molecular mechanisms underlying the loss of functional fitness in patient-derived CD8^+^ Treg cells have been uncovered and are closely related to a defect in exosome production (13) (**Figure 2**). Both, the loading of NOX2 into the exosomes and the generation of exosomes were attenuated due to the rewiring of the endosomal system. The endosomal machinery is critically involved in processes of protein quality control and is responsible for the trafficking of intracellular proteins between different cellular compartments and the release of proteins through exosomes. New insights into the different vesicular trafficking pathways have emerged, and the endosomal sorting complex required for transport (ESCRT) pathway is now recognized as the pathway for the formation of intraluminal vesicles and multivesicular bodies (MVB) (105). Exosomes are born by intraluminal budding of the MVB (106). The multiprotein complex of the ESCRT machinery regulates the invagination of vesicles into the MVB while recognizing, capturing, and sorting ubiquitinated protein cargo. Sequestering of ubiquitinated membrane proteins can occur at the endosomal membrane as well as the plasma membrane. When the MVB fuses with the plasma membrane, exosomes can be secreted into the extracellular space. Much has yet to be learned about the trafficking, docking and membrane integration of exosome carrying MVB, but several Rab GTPases within the endolysosomal trafficking machinery, including Rab27a and Rab27b, are associated with exosome loading and secretion (107). In essence, effector proteins recruited by Rab GTPases ultimately determine the collection of cargo, the movement of vesicles throughout the subcellular compartments, the docking of MVBs to the target membrane and the delivery of exosomes. In the case of CD8^+^ Treg cells derived from GCA patients, hyperactivation of NOTCH4 signaling disrupts the exosomal secretion of NOX2 through transcriptional control of Rab GTPases. Precisely, NOTCH4^hi^CD8^+^ Treg cells upregulate the expression of *RAB5A* and *RAB11A* but repress *RAB7A*, accumulating NOX2 in the intracellular compartment, including the early and recycling endosomes. GCA CD8^+^ Treg cells fail to translocate NOX2 to MVBs and the cell surface, disrupting the exosomal release of immunosuppressive NOX2 (**Figure 2**). Ultimately, dysfunctional CD8^+^ Treg cells are unable to control the expansion of pro-inflammatory CD4^+^ T cells, paving the way for the invasion of an immune privileged tissue site (13). These data will allow the development of new strategies to modulate the balance between pro and anti-inflammatory T cells in GCA. Inhibition of NOTCH signaling repaired CD8^+^ Treg function *in vitro* and *in vivo* and ameliorated vascular inflammation in NSG mice carrying human arteries (13) provide a rationale for drug targeting of this vaso-inflammatory pathway.

#### Takayasu Arteritis (TAK)

Takayasu’s arteritis (TAK) is an inflammatory vasculopathy that primarily affects young women and leads to aortitis and vasculitis of the primary aortic branch vessels (108). While the histopathology of vascular involvement and the consequences of vascular inflammation have similarities in GCA and TAK, there are also differences in the cellular and molecular immunopathology of the two vasculitides (103). The percentage of circulating activated Treg has been reported to be lower in TAK patients than in healthy age-matched controls, while resting Tregs are similar (109, 110) suggesting the possibility of abnormal Treg cell maturation. On account of their plasticity, Treg cells can acquire new effector functions, e.g. differentiating into Th1, Th2, or Th17-like cells (20). But this transformation of Treg cells can possibly strengthen the inflammatory processes and further diminish physiological immunosuppression. Another possibility based on recent studies proposes that Tregs derived from TAK patients insufficiently differentiate into Th2-like cells, thereby detracting from IL-4 and IL-13 production and contributing to excess inflammation (110).

#### Polyarteritis Nodosa (PAN)

Polyarteritis nodosa, formerly known as periarteritis nodosa, is a systemic necrotizing vasculitis affecting medium and small-sized vessels (111). Vascular damage occurs preferentially in the gastrointestinal tract, skin and peripheral nervous system. Unlike GCA and TAK, PAN patients have been reported to have increased Treg cells in their blood (112). However, in co-culture experiments with effector T cells the suppressive abilities of Tregs from PAN patients are significantly depressed, and this loss of function appears to be associated with lower expression of CTLA-4. Studies comparing Treg cell competence in patients treated with prednisolone or prednisolone plus cyclophosphamide have found normalization of Treg cell frequencies (112).

#### Kawasaki Disease (KD)

Kawasaki disease (KD) is a vasculitis of early childhood targeting medium and small-sized vessels. A classical complication of KD is the formation of coronary artery aneurysms. In the acute phase of KD, Treg cells frequency is lowered by 50% compared to age-matched healthy controls, regardless of whether the patients had coronary artery lesions (CAL) or not (113–115). The gold standard of therapy for KD is now the infusion of immunoglobulins (IVIG), which modulates abnormal immunity through the antibody-dependent pathways and increases the number of circulating Treg cells (116).

Fc-specific Treg clones have been generated from sub-acute KD patients without arterial complications following IVIG therapy. These Treg clones display an unusual phenotype, secreting IL-10 and IL-4 but not TGF-β. However, KD patients with CAL even despite IVIG treatment seem to be unable to expand these Fc-specific Treg populations (117). Similar Treg fine specificities have been isolated from IVIG-treated KD patients and healthy controls, suggesting that the absence of Fc-specific Treg cells in acutely ill KD patients may be an inflammation-imposed abnormality (118). Patients with a history of KD in their childhood did not respond to Fc protein *in vitro*, suggesting that the IVIG-induced Treg response in KD patients is short-lived. Infliximab, a chimeric monoclonal antibody targeting TNF-α, can increase Treg cell frequencies during acute KD, while Infliximab-resistant patients lack an adaptation of Treg cell frequencies (119). All of these observations support the hypothesis that an inflammatory state can alter the frequencies of CD4+ FOXP3+ cells in the circulation and can possibly strip these cells of their suppressive capabilities.

In KD, FOXP3 mRNA levels appear to be regulated by the miR-155/SOCS1 and the miR-31 signaling pathways. In patients with acute KD, decreased CD4^+^FOXP3^+^ Treg cells might be associated with decreased expression of miR-155, leading to aberrant SOCS1/STAT5 signaling and overexpression of miR-31. This abnormality may also represent an inflammation-imposed deviation as it can be corrected by IVIG (114).

#### HCV-Associated Cryoglobulinemic Vasculitis

Vasculitis associated with Cryoglobulinemia is an immune complex disease that unfolds primarily in small-sized vessels, such as the capillaries of renal glomeruli. All patients with cryoglobulinemic vasculitis require evaluation for chronic hepatitis C virus (HCV) infection (120). Early studies showed that patients with symptomatic HCV-associated cryoglobulinemic vasculitis have both reduced numbers and diminished function of Treg cells (121), consistent with the hypothesis that systemic inflammatory states are characterized by redistribution and functional impairment of Treg cells.

A French team of investigators has evaluated the effectiveness of different therapies on the Treg cell population in 3 prospective trials. PEGylated interferon alfa-2b plus ribavirin treatment induced a significant and stable increase of Treg cell frequencies compared with baseline in patients with clinical and viral remission (122). In contrast, Treg cell frequencies did not differ after treatment for non-responders or partial responders. The frequency of Treg cells was positively correlated with plasma complement levels and inversely correlated with cryoglobulin levels, again indicating that circulating CD4^+^ FOXP3^+^ T cells are a sensitive biomarker of systemic inflammation. Since Treg cells are dependent on IL-2 as a growth and survival factor, therapeutic trials have tested the potential benefit of supplementing low-dose IL-2 (aldesleukin) in patients refractory to conventional antiviral therapy and/or B cell depletion therapy. Monitoring of peripheral Treg cells following treatment with exogenous IL-2 has shown numeric and functional improvements (123). Beneficial effects and reversal of Treg deficiency have also been reported for combination antiviral therapy with sofosbuvir plus daclatasvir (124).

#### Henoch-Schoenlein Purpura (IgA Vasculitis)

Henoch-Schoenlein purpura (HSP), also known as IgA vasculitis, is the most common small vessel vasculitis in children (125). Target organs include skin, kidneys, gastrointestinal tract, and joints. Like most systemic vasculitides, HSP patients have lower numbers and weak suppressive function of Treg cells (126–129). As a reflection of a shift in the balance between pro-inflammatory effector T cells and suppressive Treg cells, the Th17/Treg ratio has been positively correlated with the erythrocyte sedimentation rate, kidney lesions, and multiorgan involvement (126). Treg cells that secrete IL-10 and TGF-β accumulate in the vasculitic tissue lesions in the kidneys (129). FOXP3 staining is preferentially localized in renal interstitial areas but does not correlate with proteinuria, serum albumin levels, and the histological classification of vasculitic involvement (130). In a recent study, miR-1-3p, miR-19b-1-5p, and miR-29b-1-5p were found to be up-regulated in peripheral blood mononuclear cells (PBMCs) of HSP patients, while miR-483-5p and miR-1246 were down-regulated. This shift was correlated with the Th17/Treg ratio (131). Again, numeric and functional alterations in peripheral blood Treg cells may simply reflect the high inflammatory status.

#### Behçet’s Disease (BD)

Behçet’s disease (BD) is an auto-immune/auto-inflammatory syndrome that can lead to vasculitis. The risk for BD is highest amongst individuals living along the Silk Road and the prototypic lesions are mucosal and genital ulcerations. BD stands out amongst the vasculitides by its often concurrent venulitis (132). Like other vasculitides, Treg cells are decreased in BD and correlate with disease activity (133–138). Imbalances in miRNA expression and excessive IL-21 have been considered to be key underlying abnormalities leading to Treg cell dysfunction (135, 138). Infliximab, but not colchicine or cyclosporine, increases the percentage of FOXP3^+^ cells in BD (134). Observation studies have reported that treated BD patients with low circulating Treg populations experience more ocular inflammation than those higher numbers (134). T cells exposed to infliximab had increased expression of FOXP3 and TGF-β and suppressed the activation of bystander T cells in *in vitro* experiments (134).

#### Antineutrophil Cytoplasmic Antibody (ANCA)-Associated Vasculitis (AAV)

AAV is a necrotizing vasculitis affecting mostly small-sized vessels. Typically, few or no immune complex deposits are seen in affected tissues. The term AVV encompasses three disease entities: granulomatosis with polyangiitis (GPA, Wegener’s granulomatosis), microscopic polyangiitis (MPA) and eosinophilic granulomatosis with polyangiitis (EGPA, Churg-Strauss syndrome) (139, 140). These three vasculitides are associated with specific autoantibodies, antineutrophil cytoplasmic antibodies (ANCA) that target either proteinase 3 (PR3) or myeloperoxidase (MPO). Each vasculitic entity has a specific phenotype with a particular pattern of organ damage. GPA is more often associated with anti-PR3 ANCAs and manifests with necrotizing granulomatous inflammation involving the upper and lower respiratory tract and necrotizing glomerulonephritis. MPA is typically associated with anti-MPO antibodies. Granulomatous inflammation is not a feature of MPA which presents with necrotizing glomerulonephritis and pulmonary capillaritis. Patients with EGPA have asthma, eosinophilia, and eosinophil-rich, necrotizing granulomatous inflammation often involving the respiratory tract. While the respiratory tract and the kidneys are targeted in most AAV patients, manifestations in the skin and the peripheral nerves are not unusual (141, 142).

Patients with AVV follow the general rule that systemic inflammation is associated with low numbers and functional impairment of Treg cells (143–146). Some studies have shown increased numbers of Treg cells (147, 148). Treg cells from GPA patients are still able to suppress proliferation of T cells from ANCA-negative patients, which has led to the hypothesis that part of the Treg deficit derives from target cell resistance (149). In AAV patients, expression of a FOXP3 isoform lacking exon 2 has raised suspicion of Treg cell instability (143). Also, miR-142-3p is upregulated in memory Tregs in GPA (150). *In vitro* overexpression of miR-142-3p produces functionally impaired Treg cells characterized by decreased cAMP levels. Pharmacological induction of cAMP production restores suppressive capacity (150). In MPA, attention has focused on the potential impact of diminished serum tryptophan and elevation of the tryptophan metabolite, kynurenine. Inhibition of tryptophan degradation enhances immune responsiveness, with a tendency to more severe glomerulonephritis (151). Correlative studies associating improvement of CCR4^+^FOXP3^+^ Treg cell frequencies with drug-free remission have supported the concept that ultimately, Treg cell frequencies are sensitive markers of the inflammatory status (152). In a mouse model, blocking IL-6 activity ameliorated the disease and increased the migration of Tregs into the kidney and the regional lymph nodes (152). In both GPA and MPA, B cell depletion therapy and conventional immunosuppressants yielded similar CD4^+^ Treg cell numbers (153).

#### Eosinophilic Granulomatosis and Polyangiitis

Patients diagnosed with Eosinophilic Granulomatosis and Polyangiitis (EGPA) typically present with the triad of asthma, eosinophilia, and necrotizing vasculitis (154). Whether eosinophils are causally involved in the damage of small blood vessels remains unresolved. Compared to asthma control patients, EGPA patients have lower frequencies of Treg cells (155, 156). Production of IL-10, TGF-β, and expression of CTLA-4 by Treg cells are correlated to the inflammatory activity of the disease (156, 157). Relapsing patients, compared to those in remission, have a lower proportion of inducible Treg cells (134) and IL-2 production is predictably low. There is some evidence that the expression of the immunosuppressive molecule indoleamine 2,3-dioxygenase (IDO) is diminished in EGPA. IDO is thought to cause immune suppression through the breakdown of tryptophan. In relapsing EGPA, Treg cells are positively correlated with the CD19^+^ B cell count and inversely related to CD80^+^CD19^+^ B cells (156). Percentages of Treg cells, Treg-derived IL-10 and TGF-β are positively correlated with the percentage of CD83^+^ dendritic cells and inversely correlated with CD206^+^ DCs (158). These data suggest that Treg cells might play a role in DC and B cell biology.

#### Rheumatoid Arthritis (RA) and Systemic Lupus Erythematosus (SLE)

As autoimmune disorders, vasculitides share features with other autoimmune diseases raising the possibility that Treg cell dysfunction is critically involved in the loss of self-tolerance. We will briefly review the current state of knowledge about Treg cell biology in the two classical autoimmune diseases, rheumatoid arthritis (RA) and systemic lupus erythematosus (SLE).

**Figure 3:**
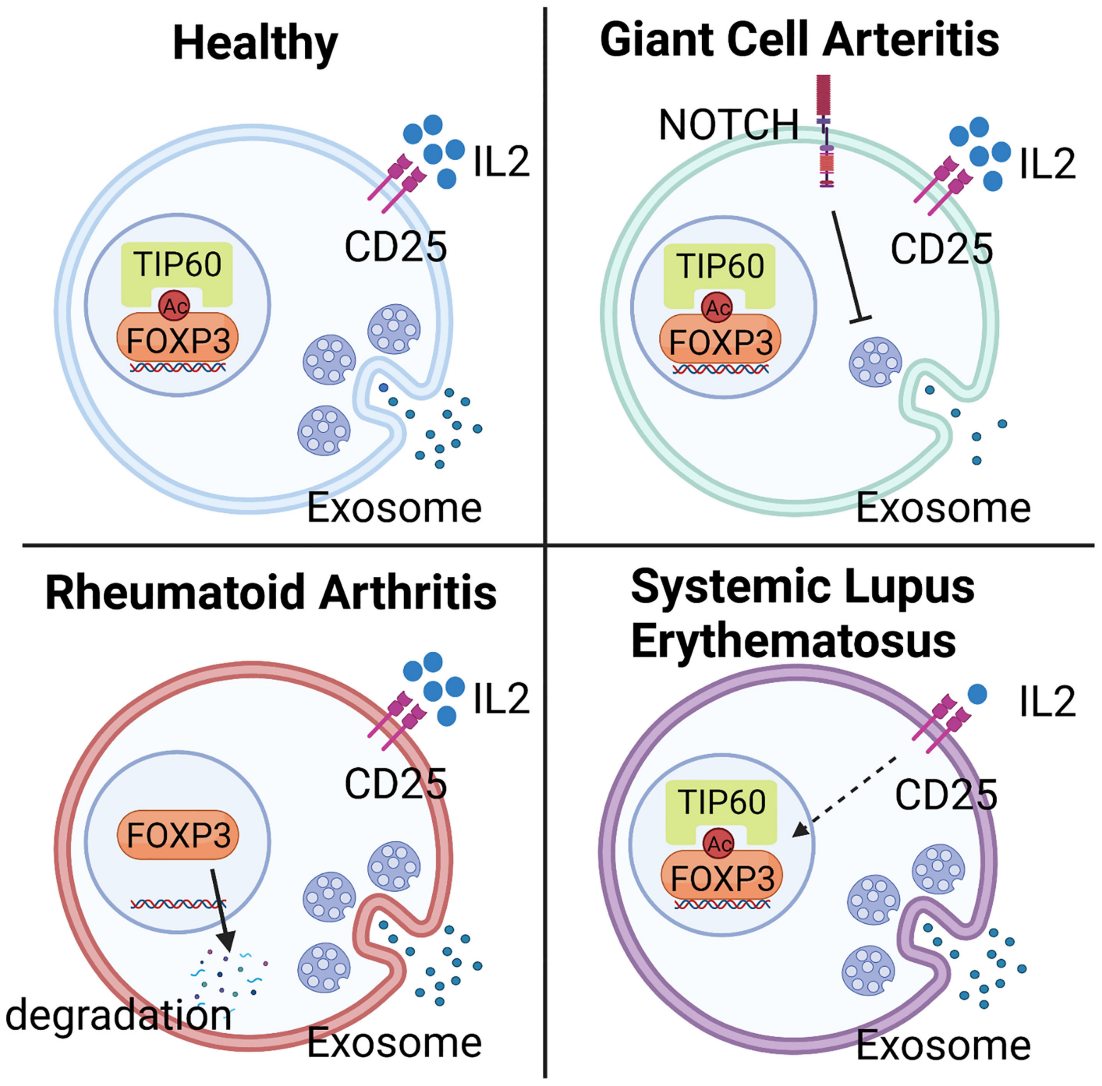
Mechanisms underlying Treg deficiency in different autoimmune diseases. Abnormalities in Treg frequency and function are observed in most autoimmune diseases, including Giant Cell Arteritis (GCA), Rheumatoid Arthritis (RA), and Systemic Lupus Erythematosus (SLE), but molecular mechanisms underlying the deficiency are disease specific. In GCA, hyperactivity of NOTCH4 signaling in CD8^+^ Treg cells reroutes the intracellular vesicle trafficking and blocks the production of NOX2-containing exosome, leading to impaired immunosuppressive function. In RA, low expression of the histone acetyltransferase TIP60 in Treg cells causes abnormal post-translational modification and instability of FOXP3, impairing Treg cell differentiation and immunosuppressive ability. In SLE, deficiency of Treg cells mainly results from the low availability of IL-2, a consequence of defective IL-2 expression by surrounding cells, such as effector T cells.

CD4^+^FOXP3^+^ T cells have been extensively studied in RA patients, but the mechanistic implications continue to be debated. Much of the discussion has focused on the methodologies of Treg analysis. One meta-analysis arrived at the conclusion that numbers of Treg cells in peripheral blood are diminished but the cells redistribute and accumulate in synovial fluid (159). Similarly, functional studies have supported the idea that systemic inflammation results in reduced numbers and reduced function. Functional analyses have shown that inhibitory competence is partially impaired. Specifically, Treg cells isolated from the peripheral blood of RA patients block the proliferation of effector T cells but fail to limit pro-inflammatory cytokine production (160). This effect can be partially reversed by anti-TNFα therapy, which successfully restores the capacity of Treg cells to inhibit cytokine production, and in parallel, supports recovery of Treg cell numbers in peripheral circulation (160). Attempts at defining the underlying mechanism have implicated reduced expression of CTLA-4 and enhanced expression of IL-6 (161, 162). Work analyzing the stability of FOXP3 has yielded valuable insights into the post-translational modification of the transcription factor as a determining feature of Treg phenotype and fitness. Specifically, loss-of-function of the histone acetyltransferase TIP60 (KAT5) has been associated with the instability of FOXP3, impaired Treg cell differentiation and failed immunosuppression (55) (**Figure 3**).

Systemic Lupus Erythematosus (SLE) is the prototypic multi-organ autoimmune disease that affects the skin, kidneys, lungs, joints, and the central and peripheral nervous systems. SLE patients follow the classical pattern and have lower frequencies of Treg cells in their blood (163). Excellent progress has been made in defining the molecular abnormalities that produce Treg cell deficiency. A prime defect of SLE Treg cells lies in the low availability of IL-2, which is required for the expansion and survival of these immunoinhibitory T cells (**Figure 3**). In SLE patients, the cAMP responsive element modulator-α (CREMα), a transcriptional repressor of the IL-2 promoter, is hyperactive, resulting in silencing of IL-2 and reduced FOXP3 expression (164). An alternative mechanism has been found for IL-2 deficiency in SLE. Mutations in the transcriptional repressor, Ikaros (165), lead to elevated protein phosphatase 2A (PP2A) expression in SLE T cells, restraining IL-2 expression (166). PP2A knockdown increased the expression of phosphorylated cAMP response element-binding protein (pCREB), restoring IL-2 expression (167). However, specific ablation of PP2A in murine Treg cells caused the development of autoimmunity in an mTOC-dependent manner (168), demonstrating that PP2A mediates different signaling pathways in effector and regulatory T cells.

### Treg Cell-Targeted Therapy

**Figure 4:**
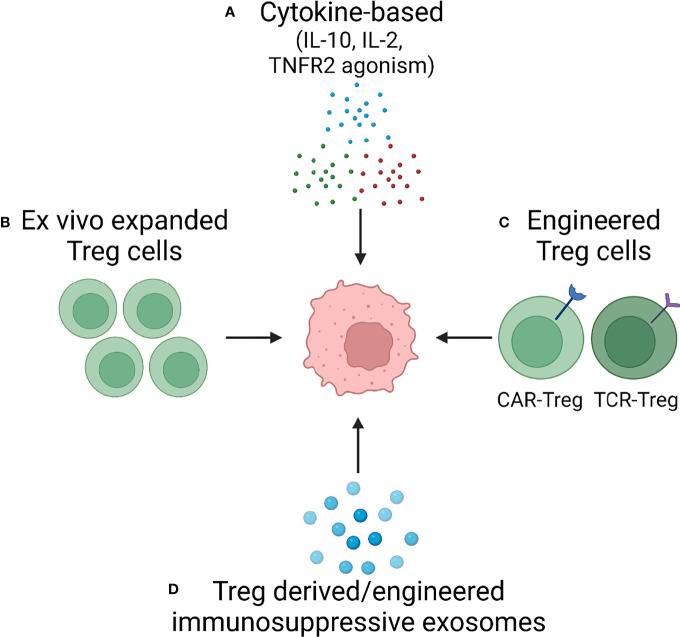
Treg cells as Therapeutic Tools – Approaches in Development. Based on increasing understanding of Treg cell biology, several approaches are in development to harness the immunosuppressive capabilities of Treg cells. Current attempts to develop versatile, effective and functionally competent cells and reagents that can be applied as immunomodulatory treatments in autoimmune disease fall into four categories: **(A)** Cytokine-based therapy applying suppressive cytokines (e.g., IL-10) or providing growth factors for Treg expansion (IL-2). **(B)** *Ex vivo* expansion of Treg cells. **(C)** Engineering of highly competent, antigen-specific or chimeric antigen receptor Treg cells. **(D)** Exploitation of exosomes, small membrane vesicles derived from multivesicular bodies and released at the plasma membrane. Treg cells package a diverse cargo into exosomes to communicate with their cellular neighbors and such exosomes can be generated as cell-free reagents for precise delivery of information to other immune cells and to tissue cells.

Targeting the immunosuppressive function of Treg cells holds great promise not only for the field of autoimmunity but also in tumor therapy, wherein excessive Treg-derived immunosuppression undermines the host’s immune response. Ultimately, the goal is to have a therapeutic armamentarium, which would allow readjustment in the numbers and the function of Treg cells *in vivo*. Below we will review the different approaches that are currently under development to exploit Treg biology for novel strategies of immunomodulatory therapy. An overview is provided in **Figure 4**.

#### Cytokine Based Therapy

##### Recombinant IL-10

The anti-inflammatory cytokine IL-10 is one of the most important immuno-suppressive molecules that Treg cells secrete. Studies have explored the potential of recombinant IL-10 as a treatment for autoimmune disease, most extensively in rheumatoid arthritis (RA) (169). In a clinical trial, IL-10 treatment of RA patients was able to suppress the key pro-inflammatory cytokines TNFα and IL-6. However, IL-10 also led to B cell activation and subsequent autoantibody production, which has been a limiting side effect (170).

##### Low Dose of IL-2

A prototypic biomarker of Treg cells is the constitutive surface expression of CD25, the alpha-chain of the IL-2 receptor, which binds the cytokine with low affinity. Interleukin-2 (IL-2) is the main cytokine supporting Treg cell development, survival, and suppressive activity. Treg cells cannot supply their own IL-2 but depend on exogenous IL-2. Hence, there is a strong rationale to utilize IL-2 supplementation to improve Treg cell function. IL-2 not only binds to the alpha chain but with even high affinity to beta and gamma chain complexes expressed on memory T-cells and NK cells. Therefore, IL-2 supplementation could have pro-inflammatory effects. However, low-dose IL-2 preferentially targets Treg cells with high CD25 expression, providing a favorable window for IL-2 treatment in autoimmune disease (171). There is some support that this may be occurring in patients. In HCV-induced vasculitis patients, “ultra-low” doses of IL-2 induced expansion of CD4^+^CD25^+^FOXP3^+^ Tregs, while effector T-cells appeared relatively unaffected, encouraging the use of IL-2 as a therapeutic strategy in other vasculitides (123).

##### TNF Receptor Agonism

Tumor necrosis factor receptor 2 (TNFR2, CD120b) is one of the receptors for the pro-inflammatory cytokine, TNFα, which is a critical mediator in many autoimmune diseases. Unlike TNFR1, which exhibits pro-inflammatory effects, TNFR2 is considered to have an anti-inflammatory function and is highly expressed on Treg cells. Activation of TNFR2 enhanced the expansion and function of Treg cells (172), suggesting the possibility of using TNFR2 agonism for immunomodulatory therapy of autoimmune diseases. In purified T cells from patients with type1 diabetes, TNFR2 agonism successfully killed autoreactive CD8^+^ T cells, but not CD4^+^ T cells (173). Targeting TNFR2 on Treg cells awaits translation into the clinic.

#### Cell-Based Therapy

##### Ex Vivo Expanded Treg Cells

Basically, cell-based therapy with Treg cells relies on the extraction, *ex vivo* expansion, activation, and reinjection of autologous Treg cells back into the patient to restore the balance between pro and anti-inflammatory immune cells.

Because of its relatively low technical challenges, *ex vivo* expanded Treg cell therapy has been the first Treg-targeted strategy in clinical trials. A series of clinical trials have been performed, exploring Treg replacement in different autoimmune diseases. One clinical trial in type 1 diabetes mellitus (T1DM) patients reported potential improvement in the longevity of pancreatic islets. Also, excellent disease control in some patients without severe side effects has been reported (174). A major concern about the transfer of *ex vivo* expanded Treg cells is the risk of significant immune repression producing compromise of host defense against exogenous pathogens and malignancies. To date, these concerns remain theoretical. In one clinical trial of SLE patients, *ex vivo* educated Treg cells traffic to and accumulate in inflamed tissue sites, where IFNγ and IL-17 expression was successfully suppressed (175). Overall, there remains optimism that *ex vivo* expanded Treg cells could be developed into a powerful therapeutic tool, reestablishing tissue tolerance in autoimmune diseases. It has also been proposed that the efficacy of transferring *ex vivo*-expanded Treg cells could be enhanced by combining cell-mediated therapy with cytokine activation therapy, e.g., IL-10, IL-2, or TNFR2 agonism. One limitation of this approach is centered on the observation that the Treg cells grown in tissue culture become addicted to IL-2, thereby jeopardizing their survival following transfer into an IL-2-poor environment. A remedy may lie in genetically engineered transferred Treg cells programmed to make their own IL 2.

##### Engineered Antigen-Specific Treg Cells (TCR-Treg)

Ideally, interference with Treg function would be targeted to the specific autoantigens driving autoimmunity. To achieve this goal, Tregs can be engineered with a predetermined antigen-specificity by transfecting them with a viral vector encoding a specific T cell receptor (TCRs). Whereas polyclonal Treg cells with unknown antigen specificities can induce unwanted effects, specifically, systemic immunosuppression, using antigen specific Treg cells could avoid this side effect. Also, antigen specific Treg cells have been found to outperform polyclonal Tregs in terms of immunosuppression. In a model system of murine T1DM, a low number of TCR-Tregs could successfully prevent and even reverse the disease (176). The limitation of this therapy lies in the difficulty of identifying relevant autoantigens. In the case of tumor cells, shared antigens, such as CD19 can be used to target engineered T-cells to a specific site. To date, no autoantigens have been used in clinical trials to suppress inappropriate immunity in autoimmune disease because the design of a high-affinity autoantigen-specific TCR to be transduced into Treg cells has remained a challenge. Single-cell sequencing applied to identify relevant TCRs may overcome some of these technical obstacles. Harnessing this technique, thousands of TCR sequences from sites of autoimmune tissue inflammation could be identified to create personalized and specific Treg cells for patients with difficult-to-manage autoimmune disease (177–179).

##### Engineered Chimeric Antigen Receptor Treg (CAR-Treg) Cells

Analogous to CART cells in tumor therapy, Treg cells can be efficiently engineered with a predetermined antigen-specificity by transfecting them with chimeric antigen receptors (CARs). CARs typically possess a single-chain variable fragment, usually a binding moiety of a monoclonal antibody, combined with an extracellular hinge, a transmembrane region, and, finally, an intracellular signaling domain. The major advantage of CAR-modified Tregs cells is that they can be engineered in a non-HLA restricted manner and are therefore broadly applicable. Compared to the wild-type TCR intracellular domain, the chimeric TCR intracellular domain has a higher capacity to activate T cells without co-stimulation and CAR-Treg cells are believed to require less IL-2 for long-term survival. Thus, the introduction of CARs into Treg cells provides both additional antigen specificity and the required signals to fully activate Treg cells and exploit their suppressive activity (180). Conversely, enhanced receptor signaling equips CAR-Treg cells with greater activity than polyclonal or TCR-Treg cells but may lead to too much immunosuppression. If the targeted autoantigens are not solely expressed in the inflamed site, the intense activation signaling of CAR-Treg cells may have avoidable side effects and this has to be weighed against the modest suppressive capacity of TCR-Treg cells. Also, the independence of CAR-Treg cells from co-stimulation may turn them into aggressive suppressors and their inherited strengths of activation may make them more likely to reach exhaustion. So far, CAR-Treg cells have not been used in humans, but promising CAR-Treg therapies have been reported in animal models of transplantation and autoimmunity (181). Human T cells engineered with a chimeric antigen receptor were able to eliminate autoantigen-specific B cells in pemphigus vulgaris (182).

#### Exosomes as Potential Immunosuppressive Tools

Exosomes are nanosized extracellular vesicles (EV) that originate in the endosomal system and are secreted to the extracellular space when multivesicular bodies fuse with the cell membrane. As lipid bilayer membrane-enclosed vesicles, exosomes are a heterogeneous population, able to transport and deliver a multitude of proteins and nucleic acids. Exosomes are taken up by recipient cells, thus imposing strong immunomodulatory effects. Release of exosomes is one of the major mechanisms through which Treg cells communicate with surrounding cells (**Figure 1**). CD8 Treg cells function by secreting NOX2-containing exosomes that then suppress membrane-proximal TCR signaling in nearby CD4^+^ T cells. The loss of NOX2-rich exosomes defines the loss of Treg activity in GCA (12, 13). In recent years, the concept of replacing dysfunctional Treg cells by transferring exosomes has attracted attention, as it might be possible to harness these vesicles for the therapeutic delivery of RNAs, peptides, proteins, and synthetic drugs. As cell-free reagents, therapeutic exosomes have numerous advantages, but challenges remain in achieving proper targeting and delivery. Some progress has been made in realizing the idea of therapeutic exosomes. One example are exosomes derived from anti-tumor CAR-T cells. Such exosomes were capable of attacking cancer cells in a CAR-T cell-free manner (183).

Methods have been developed to produce exosomes or exosome-like nanoparticles with defined payloads and progress is being made in designing ways of delivering the microvesicles (MV) to specific cells, thus increasing local concentrations and minimizing systemic side effects. Given the potent immunosuppression imposed by NOX2-containing exosomes, NOX2-loaded MV or exosome-like nanoparticles may provide the means to mimic Treg function *in vivo*. This approach would offer numerous advantages: (1) Better access to inflamed tissue sites because of the much smaller size of exosomes compared to Treg cells; (2) Potentially higher suppressive capacity. Treg cells need to use most of their energy and biosynthetic molecules for cellular maintenance, but artificial exosomes containing only their payload can deposit high local concentrations of suppressive proteins; (3) Easier control of unintended systemic immunosuppression. In cell-based Treg therapy, it is impossible to completely remove transduced Treg cells when generalized immunosuppression becomes a problem. In contrast, exosomes are short-lived. (4) Tight management of suppressive ability. Both cytokine and Treg therapy rely on the response of the immune cells in the host to produce the ideal effect. In patients that are immunocompromised, this goal may be difficult to reach. However, exosomes can directly manipulate the targeted cell population, thereby avoiding the intermediate steps, bringing about a more controllable result; (5) Low risk of contamination and rapid turnaround for production. *Ex vivo* cell engineering is demanding, complicated by possible contamination with pathogens and is a very time-consuming process. Production of exosomes can be achieved under industrialized conditions, can provide therapeutic reagents in a short time, with a low badge to badge variability; (7) Finally, exosomes can be designed to be independent of HLA restriction and could thus be used in the majority of patients.

### Summary and Conclusions

Autoimmune vasculitides are a heterogeneous group of disorders that share in common that immune-mediated processes damage blood vessels, almost always capillaries, arterioles, and arteries. In some of the vasculitides, vascular injury results predominantly from T-cell dependent pathways, in others, autoantibodies participate in vessel wall destruction. The age range at disease onset is broad, most patients require aggressive immunosuppressive therapy and some of the disease manifestations are fatal or associated with severe organ failure. Like in most autoimmune diseases, how self-tolerance is lost remains enigmatic. Hence, disease management is limited to broad and nonspecific suppression of host immunity, associated with a high risk to compromise protective immunity against cancers and infections. New therapeutic approaches in managing these chronic and destructive autoimmune diseases are urgently needed.

Available data indicate low numbers and defective function of circulating Treg cells in most patient populations. The common denominator appears to be loss or redistribution of Treg cells, a phenomenon shared with other autoimmune diseases (**Table 1**). Given the diversity of target tissues, pathomechanisms and immune cell abnormalities in the vasculitides, reduction in circulating Treg cell numbers and their functional impairment are almost certainly a consequence of systemic inflammation. Accordingly, Treg cell numbers, phenotypes and *ex vivo* functional competence often improve with immunosuppressive therapy. Dynamic changes in Treg cell numbers and fitness argues against intrinsic defects in the patients’ Treg cells and supports the concept that the inflammatory environment critically shapes Treg cell survival, trafficking and competency.

**Table 1:** Treg Dysfunction in Autoimmune Vasculitis.

Vasculitis	Treg Phenotype		Molecular Mechanisms
	Frequency	*Ex vivo* Function	
GCA	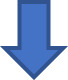	impaired	aberrant NOTCH4 signaling rerouted trafficking of intracellular vesicles suppressed formation of multivesicular bodies suppressed exosome biogenesis
TAK	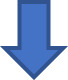 activated Treg	impaired loss of Th2-like Treg cells	
PAN	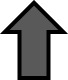	impaired loss CTLA-4 expression	
KD	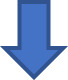	impaired lack of Fc-specific Treg	decreased miR-155, increased miR-31 altered SOCS1/STAT5 signaling
Cryoglobulinemic Vasculitis	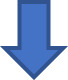	impaired	
HSP	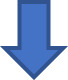	impaired shifted Th17/Treg ratio	up: miR-1-3p, miR-19b-1-5p, miR-29b-1-5p, down: miR-483-5p miR-1246
BD	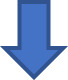	impaired excessive IL-21	dysbalanced miRNA expression
AAV	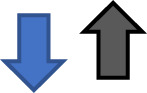	impaired	GPA: FOXP3 lacking exon 2 GPA: upregulated miR-142-3p MPA: diminished serum tryptophan
EGPA	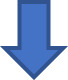	impaired low CLTA-4, IL-10, TGF-β	diminished IDO

Arrow up, upregulated in patients; arrow down, downregulated in patients.

Detailed information on the molecular mechanisms underlying Treg cell dysfunction in vasculitides is mostly lacking, with the exception of giant cell arteritis, in which loss of function of immunosuppressive exosome production has been attributed to the rerouting of intracellular vesicles (**Table 1**). Precisely, patients’ CD8 Treg cells aberrantly express the NOTCH4 receptor and excessive NOTCH signaling leads to a defect in the formation of multivesicular bodies (MVB), thus disrupting the production and release of immuno-inhibitory exosomes. Such exosomes are loaded with NOX2 and are highly efficient in controlling the responsiveness of CD4 T cells and the overall size of the CD4 T-cell compartment.

It is possible that some of the vasculitides share abnormalities in Treg cell function with other autoimmune diseases, particularly, RA and SLE (**Figure 3**). Lack of interleukin 2, the most important cytokine in Treg cell generation, expansion and survival is now recognized as a disease mechanism in SLE (**Figure 3**). Here, exogenous IL-2 emerges as a potential therapeutic intervention. In RA, instability of the lineage-determining transcription factor FoxP3 renders Treg cells short-lived and dysfunctional (**Figure 3**). Mechanism-oriented investigation will be needed to uncover the pathways that cause Treg cell loss-of-function in each of the vasculitides. Progress in the field will require turning towards molecular explorations of relevant Treg populations in secondary lymphoid tissues as well as in the disease lesions.

The major appeal of increasing the knowledge of Treg cell biology in the vasculitides derives from the potential to translate such knowledge into new and molecularly defined therapeutic interventions. Multiple options exist, all exploiting the key mechanisms through which Treg cells impose their immunoregulatory capacity (**Figure 4**): treatment with inhibitory cytokines; supplementation of IL-2; cell-based therapy transferring *ex vivo* expanded or appropriately engineered Treg cells and, finally, replenishing immune-suppressive exosomes. Participation of exosomes in the induction and maintenance of self-tolerance emphasizes their potential to replace Treg cells in autoimmune disease. They exhibit desirable features, such as a high delivery efficiency, a long circulating half-life, an intrinsic ability to target tissues, they are biocompatible and have minimal toxicity. Appropriate clinical trials need to test the applicability of Treg derived exosomes *in vivo*.

## Author Contributions

KJ, SP, and CW selected the review topic, collected relevant publications, designed the Figures and wrote the review. GB, KW, and JG made conceptual contributions and participated in writing. All authors contributed to the article and approved the submitted version.

## Funding

This work was supported by the National Institutes of Health (R01 AR042527, R01 HL117913, R01 AI108906, R01 HL142068 to CW and R01 AI108891, R01 AG045779, U19 AI057266, R01 AI129191 to JG. SP received fellowship support from the Servier Institute.

## Conflict of Interest

The authors declare that the research was conducted in the absence of any commercial or financial relationships that could be construed as a potential conflict of interest.

## Publisher’s Note

All claims expressed in this article are solely those of the authors and do not necessarily represent those of their affiliated organizations, or those of the publisher, the editors and the reviewers. Any product that may be evaluated in this article, or claim that may be made by its manufacturer, is not guaranteed or endorsed by the publisher.
